# Frequency of Hepatitis B Virus infection among Patients before Chemotherapy Treatment

**DOI:** 10.31557/APJCP.2021.22.9.2939

**Published:** 2021-09

**Authors:** Shahab Mahmoudvand, Somayeh Shokri, Habibollah Mirzaei, Ali Ramezani, Manoochehr Makvandi, Niloofar Neisi, Sayed Jalal Hashemi

**Affiliations:** 1 *Infectious and Tropical Disease Research Center, Health Research Institute, Ahvaz Jundishapur University of Medical Sciences, Ahvaz, Iran. *; 2 *Department of Virology, School of Medicine, Ahvaz Jundishapur University of Medical Sciences, Ahvaz, Iran. *; 3 *Research Institute for Infectious Diseases of the Digestive System, School of Medicine, Ahvaz Jundishapur University of Medical Sciences, Ahvaz, Iran. *

**Keywords:** Occult Hepatitis B, chemotherapy, enzyme-linked Immunosorbent Assay (ELISA), nested-PCR

## Abstract

**Background:**

Hepatitis B virus (HBV) is an important public health problem worldwide. Chronic HBV in patients undergoing chemotherapy and immunosuppressive treatment are at risk of HBV reactivation. The consequence of HBV reactivation in immunosuppressed patients may lead to liver failure and death. Therefore, this study was conducted to investigate the frequency of HBV markers in cancer patients before chemotherapy.

**Materials and Methods:**

In this study cross-sectional, blood samples were collected from 90 cancer patients before chemotherapy. The patient’s sera were tested for the presence of HBsAg and anti-HBc using enzyme-linked immunosorbent assay (ELISA). The HBVDNA was tested for patient’s sera using nested polymerase chain reaction (nested-PCR).

**Results:**

Among 90 patients, 42(46.7%) were males and 48 (53.3%) females, with a mean age of 52.52 ± 11.71 years (range, 25–83 years). Of the 6/90 (6.66%) patients, including 4/42 (9.5%) males and 2/48 (4.1%) females cases were positive for HBsAg, anti-HBc and HBV DNA, (P=0.31). The frequency of HBV infection in cancer patients was rectal 3(3.33%), breast cancer 2 (2.22%) and prostate 1(1.11%) cases. The sera of 8/84 (9.52%) patients including 5/39 (12.82%) males and 3/45 (6.66%) females tested positive for anti-HBc, but negative for HBsAg and HBV DNA. (P=0.55). The results of phylogenetic tree revealed that four isolated HBV DNA in cancer patients were cluster with genotype D.

**Conclusions:**

High frequency of 6.66% HBV infection have been observed in cancer patients before chemotherapy. The sera of 9.52% patients were only positive for anti-HBc IgG which may indicate the past HBV infection or presence of OBI but requires further investigation. To prevent HBV or OBI reactivation, the screening of HBV DNA and anti HBc should be implemented for cancers patients before chemotherapy.

## Introduction

Chronic Hepatitis B infection is the major concern public health problem worldwide. It has been estimated more than 240 million of world population are living with chronic HBV infection ( Hayat Davoudi et al., 2020; Thu et al.,2019; Khazaei et al 2018). The persistence replication of HBV in hepatocytes may result in wide spectrum of liver disease such as cirrhosis, fibrosis and hepatocellular carcinoma (Liang et al., 2009). HBV reactivation was observed in cancer patients under chemotherapy which lead to liver failure and death (Pattullo.,2016;Tavakolpour et al.,2016). HBV reactivation is defined serum HBV DNA levels raising at least 10 times (relative to baseline) and ( ALT level ≥3-fold increase compared to baseline and >100 U/L). (Lu et al 2017; Patel et al 2015; Paul et al.,2016).

Reactivation of occult hepatitis B infection (OBI) was exhibited in cancer patients underwent chemotherapy which resulted in fulminant hepatitis (Squadrito et al., 2014). OBI is defined by the detection of HBV DNA liver tissue/or in serum of HBsAg negative subjects and the level of HBV-DNA in the serum is usually low (< 200 IU/mL/or 1,000 copy number/mL) (Kwak et al., 2014). Two classes of OBI were described, seropositive and seronegative. Seropositive OBI refers to the presence of anti-HBc and/or anti-HBs in the serum but HBV DNA is detected in serum/liver. While seronegative OBI refers to when both anti-HBc and anti-HBs are negative in the serum but HBV DNA is detected in serum/liver (Makvandi, 2016). Recently it was shown that detection of anti-HBc alone in the blood may be used as a surrogate marker to identify OBI (Raimondo et al., 2018). Similarly, anti-HBc was detected in some blood donors with OBI who had undetectable HBV DNA in mini-pool NAT (nucleic acid testing) (Spreafico et al., 2015). HBV reactivation has been reported in individuals negative HBsAg, positive anti-HBc who had undetectable HBV DNA in the blood (Cholongitas et al., 2018). OBI reactivation occurs in patients with hematological malignancies, undergoing immunosuppression, organ transplant, underwent and chemotherapy (Masarone et al .,2014; Iloeje et al., 2010). The detection of OBI can be achieved by highly sensitive methods such as real-time PCR and nested-PCR. (Raimondo et al,.2008; Sosa-Jurado et al., 2018). 

Since, limited data is available regarding the assessment of molecular and serological HBV markers in cancer patients in Ahvaz city ,Iran therefore, this investigation was contacted to estimate frequency of HBV markers in cancer patients before chemotherapy treatment.

## Materials and Methods

*Study population and sample collection*

In this cross-sectional study, 90 patients with solid cancer before chemotherapy were selected at the clinical oncology ward in Golestan Hospital in Ahvaz, Iran during June to September 2019. The inclusion criteria: The confirmed histologically by pathologist. The patients who have not received any chemotherapy, radiotherapy anti CD20 treatment or any other immunosuppression drugs. The exclusion criteria: cancer patients with history of rheumatoid arthritis, celiac disease, liver cirrhosis, tumor discectomy, a history of HIV infection, intravenous drug abusers, and existence of other immunodeficiency diseases. 5 ml blood samples were collected from patients and then the sera stored at -80°C until further analyses. None of the patients have received HBV vaccine.

The present study with registration number IR.AJUMS.REC.1394.189 was approved by the Ethic Committee of Ahvaz Jundishapur University of Medical Sciences, Ahvaz, Iran. In addition, the informed consent was obtained from all patients. 

*Serological and liver function tests*

Serological markers (HBsAg and anti-HBc) were measured by ELISA (Diapro, Milano, Italy), according to the manufacturer’s protocols. The sera of patients positive [HBsAg,anti-HBc ] and sera samples negative HBsAg but positive anti-Hbc were further tested for HBV DNA by nested PCR. The liver function test including Aspartate Aminotransferase (AST) and Alanine Aminotransferase (ALT) were evaluated for each patient.

*DNA extraction*

DNA was extracted from sera samples using High Pure Nucleic Acid Kit (Roche Applied Science, Germany) according to manufacturer’s instructions. The extracted DNA was stored at -20°C until further use.

*Detection of HBV DNA using nested-PCR*

Detection of HBV DNA was performed by nested-PCR using the following primers: Outer primers, forward: 5′-GAGTCTAGACTCGTGGTGGACTTC-3′ and reverse: 5′-AAATKGCACT AGTAAACTGAGCCA-3′ Inner primers, forward: 5-′CGTGGTGGACTTCTCTCAATTTTC-3′ and reverse: 5′-GCCARGAGAAACGGRCTGAGGCCC-3′ (Makvandi et al., 2014). Reaction mixture and thermal cycling programs was as follows: For the first round of PCR, the PCR reaction mixture contained 1.5mM Mgcl_2_, 10x PCR buffer (Roche, Germany), dNTP mix (10 Mm) 0.5 µL (Roche, Germany), 10 pmol of each primers, and 1 U of Taq DNA polymerase (Roche, Germany) and D/W up to 25 µl . All the tests were subjected to thermocycler (Techne TC-5000, UK) with following conditions: the first round was followed with thermal condition 94°C for 5 min following 30 cycles of 94°C for 45 sec, 56°C for 30 sec and 72°C for 30 sec and final extension at 72°C for 5 min. The second round was carried out using inner primers with the same conditions as the first round. PCR product was electrophoresed in 2% agarose gel with DNA safe Stain (CinnaGen, Iran) and visualized using a UV transilluminator (Kiagen, Tehran, Iran). The PCR product of 417 bp showed the positive reaction (Makvandi et al., 2014)

*Sequencing and Phylogenic Analysis*

The four positive PCR products were sequenced (ABI sequencer, Bioneer Inc., South Korea). The results of alignment sequencing of the detected partial HBV DNA by NCBI and the Hepatitis B Virus database. The results of sequencing of the isolated HBV strains were aligned with available HBV sequences in the GenBank. MEGA 6 was applied to construct the phylogenetic tree by the maximum-likelihood. The statistical significance of the phylogenetic tree was tested by the bootstrap method (1,000 replicates) (Studier et al.,1988).

*Statistical analysis*

The mean and standard division was used for analysis patient’s age and liver function tests. The analysis of distribution of HBV markers among the sex was carried out by chi-square. Data were analyzed by Statistical Package for the Social Sciences Statistical Package for the Social Sciences 16.0 (SPSS Inc., Chicago, IL, USA) . A P value <0.05 was considered statistically significant.

## Results

Of the 90 patients 42 (46.7%) were males and 48 (53.3%) females with a mean age of 52.52 ± 11.71 years (range, 25–83 years). Among the patients, the most type of cancer was breast tumor 27 (30%), followed by rectal 17 (18.9%) and cervix 9 (10%) cancers. Six Of the 90 patients (6.6%) cases were positive for HBsAg ,anti-HBc and HBV DNA, including 4/42 (9.5%) males and 2/48 (4.1%) females (P=0.31). The frequency of HBV infection was in patients with rectal 3/17(17.6%)), breast cancer 2 2/27(7.4%) and prostate 1/8(12.5%) cases (Table-1).The cancer patients including Ovarian 5 (5.5%), Colon 4(4.4%), Bladder 3 (3.3%) Liver 3 (3.3%) Stomach 3 (3.3%) Pancreatic 2 (2.2%) Gallbladder 1 (1.1%) Parotid 1 (1.1%) Kidney 1 (1.1%) Larynx 1 (1.1%) Nasopharynx 1 (1.1%) Sinus 1 (1.1%) Testicular 1 (1.1%) were negative for HBV markers. Overall, 76/90 (84.44%) the cancer patients sera of were negative for HBV markers.

The patients were positive for positive HBV markers [ HBsAg, anti-HBc, HBV DNA] had mean upper limited normal [ALT (37.50±9.84 (U/L) and AST (42.50±6.95 (U/L)] level and patients were negative HBV markers had normal range of [ALT, 23.04±9.96 (U/L) and AST 23.96±13.10 (U/L)] levels.

Of the remaining 84 sera, 8(9.52%) cases including 5/39 (12.82%) males and 3/45 (6.66%) females tested positive for anti-HBc, but negative for HBsAg and HBV DNA. (P=0.55). The patients were only positive anti-HBc had mean [ALT (29.75±14.29 (U/L) and AST (32.16±15.10 (U/L) levels ] and patients were negative anti-HBc had mean [ALT, 22.06±9.96 (U/L) and AST (23.96±13.10U/L)] level (Table 2). Table 2 shows the prevalence of anti HBc among the males and females was not significant (p=0.55). 

**Figure 1:**
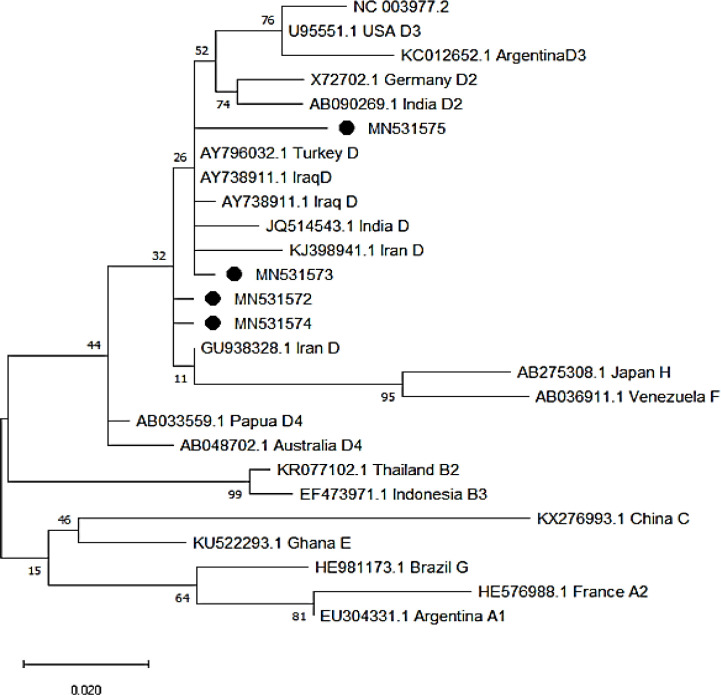
The Phylogenetic Tree with Maximum- Likelihood method was constructed with four partial sequences (S-Gene) of isolated HBV DNA strains from Ahvaz city with accession number (MN531572 to MN531575) compared with different HBV genotypes with relevant accession numbers retrieved from Gene Bank isolated from different regions of the world. The isolated HBV DNA with black circle were cluster with HBV genotype D isolated from different regions of the world. The accuracy of phylogenetic tree was assessed by 1000 bootstrap replicates. Scale bar=0.020

The frequency of positive anti-HBc was observed in cancer patients with breast 1/27 (3.7%), Rectal 2/17 (11.7%), Cervix 2/9 (22.2%), Prostate 1/8 (12.5%) and lung 2/2 (100%) cases. The sequences of four positive HBV DNA samples with accession numbers, MN531572 to MN531575 were deposited in GenBank. The analysis of phylogenetic tree shows four isolated HBV DNA with accession numbers (MN531572 to MN531575) were cluster with HBV DNA genotype D isolated from Iran (KJ398941.1 and GU938328.1) and other regions of the world (Figure 1).

**Table 1:** The Frequency of HBV Markers among the Patients with Different Types of Cancer

Cnacer Type	"Positive HBsAg,Anti HBc, HBVDNA "	"Negative HBsAg and HBVDNA , Positive Anti HBc,"
Breast	2/27 (7.4%)	1/27 (3.7%)
Rectal	3/17 (17.6%)	2/17 (11.7%)
Cervix	-	2/9 (22.2%)
Prostate	1/8 (12.5%)	1/8 (12.5%)
Lung	-	2/2 (100%)

**Table 2:** Demographic and Laboratory Characteristics Cancer Patients Only Postive and Negative for Anti-HBc

Parameter	Total	Positive Anti -HBc	Negative Anti-HBc
Age	-	61.25±12.70	51.02±11.28
Gender			
Male	39 (46.6%)	5 (7.3%)	34 (92.7%)
Female	45 (53.4%)	3 (2.1%)	42 (97.9%)
Laboratory Parameters			
ALT (IU/L)	-	29.75±14.29	22.06±9.96
AST (IU/L)	-	32.16±15.10	23.96±1310

Table 2 shows the prevalence of anti HBc among the males and females was not significant(p=0.55)

## Discussion

Cancer patients undergoing chemotherapy are at high risk of HBV/OBI reactivation and consequence of liver failure and death (Loomba et al., 2017; Coppola et al., 2011). The HBV reactivation have been reported in patients with breast, colorectal and prostate cancers (Lu et al., 2017; Patel et al., 2015; Paul et al., 2016).

In the present work 6/90 patients (6.6%) cases were positive for HBsAg, anti-HBc and HBV DNA, including 4/42 (9.5%) males and 2/48 (4.1%) females (P=0.31). The frequency of HBV infection was in patients with rectal 3/17(17.6%), breast cancer 2/27(7.4%) and prostate 1/8(12.5%) cases. The frequency of HBV markers have been reported in patients with breast , colorectal and prostate cancers (Lu et al., 2017; Patel et al., 2015; Paul et al., 2016).

The HBV reaction has been reported patients with colorectal cancer, breast cancer , prostate and cervix (Okagawa et al., 2014; Yeo et al., 2003; Yamauchi et al., 2020; Dimond et al., 2020). In this study all the isolated HBV DNA were genotype D. The analysis of phylogenetic tree shows four isolated HBV DNA with accession numbers (MN531572 to MN531575) were cluster with HBV DNA strains genotype D isolated from Iran (KJ398941.1 and GU938328.1) and from other regions of the world.

The patients were positive for positive HBV markers [ HBsAg, anti-HBc, HBV DNA] had mean upper limited normal [ALT (37.50±9.84 (U/L) and AST (42.50±6.95 (U/L)] level and patients were negative HBV markers had normal range of [ALT, 23.04±9.96 (U/L) and AST 23.96±13.10 (U/L)] levels. 

The patients were only positive anti-HBc had mean [ALT (29.75±14.29 (U/L) and AST (32.16±15.10 (U/L)] levels and patients were negative anti-HBc had mean [ALT, 22.06±9.96 (U/L) and AST (23.96±13.10U/L)] levels. Therefore, the cancer patients with HBV markers when receiving chemotherapy, are at risk of HBV reactivation. 

The sera of 8/84(9.52%) patients including 5/39 (12.82%) males and 3/45 (6.66%) females tested positive for anti-HBc, but negative for HBsAg and HBV DNA (P=0.55). It is well-known that anti-HBc alone is a predictive signal of potential OBI and it could be used as a surrogate marker to identify OBI (Raimondo et al., 2018; Roman, 2018). However, detection of HBV DNA in liver biopsy is a gold standard test to predict the OBI (Raimondo et al., 2019). Likewise , anti-HBc was detected in some blood donors with OBI with undetectable HBV DNA in their sera (Spreafico et al., 2015). In the present study the frequency of anti-HBc was observed in cancer patients with breast 1/27 (3.7%), Rectal 2/17 (11.7%), Cervix 2/9 (22.2%), Prostate 1/8 (12.5%) and lung 2/2 (100%) cases. Therefore, to predict whether the mentioned patients infected with OBI it requires HBV DNA detection in the liver biopsy which is not readily accessible. In addition to, these patients only positive anti–HBc are at risk of HBV reactivation when receiving chemotherapy. 

The presence of OBI has been detected in cancer patients with lymphocytic leukaemia hepatocellular carcinoma (HCC) and hematologic and solid cancer (Laurenti, 2015; Lok et al., 2011; Baghbanian et al., 2016.). 

The presence of OBI (positive anti HBc and high level HBV DNA) was detected in 1.4% of the 213 patients with solid cancers underwent chemotherapy, in Iran (Meidani et al., 2016). In another study conducted in Egypt, of 72 patients with diffuse large B-cell lymphoma (DLBCL) negative for HBsAg, the OBI reactivation followed by chemotherapy treatment was found in 5/10 (50%) patients positive anti-HBc ( Elbedewy et al., 2015). 

Several factors involve in HBV reactivation in overt or occult HBV infection. HBV/or OBI in HCV patients under treatment direct acting anti-virals (DAAs), chemotherapy, or immunosuppressive therapy in some solid tumors and leukemia patients under treatment with prednisolone and imatinib (Guo et al., 2018; Liu et al., 2018). Thus early identification of HBV reactivation is essential to start antiviral therapy (Kusumoto et al.,2011; Manzano-Alonso et al., 2011). The use of nucleoside analogues, lamivudine (LAM), entecavir (ETV), tenofovir (TDF), or tenofovir alafenamide (TAF) has been proved to be efective for the prevention of HBV reactivation (Reddy et al., 2015; Pisaturo et al., 2018).

The HBV immunization is strongly recommended for cancer patients ngative for HBsAg, and anti-HBc undergoping chemotherapy (Ariza-Heredia et al., 2015) The HBV vaccine response rate was found more than 70% of cancer patients receiving chemotherapy (Weitberg et al., 1985), In this study, none of cancer patients have received HBV vaccine

In conclusion, High frequency of 6.66% HBV infection have been observed in cancer patients before chemotherapy. The sera of 9.52% patients were positive only for anti-HBc IgG which may indicate the past HBV infection or presence of OBI but requires further investigation. To prevent the HBV or OBI reactivation, the screening of HBsAg, HBV DNA, anti HBc and HBV viral load should be implemented for cancers patients before and during chemotherapy treatment . HBV vaccination is recommended when HBV status of patient (HBsAg, anti-HBsAb) is negative.

## Author Contribution Statement

Shahab Mahmoudvand PCR experiment. Somayeh Shokri ELISA experiment., Habibollah Mirzaei performs the sample identification. Ali Ramezani performs the sample collection, Manoochehr Makvandi worked on the manuscript analysis and supervised the project. Niloofar Neisi performs sample identification, Sayed Jalal Hashemi. in aided in the implementation of work.
